# Dr. Q. Gibbon’s Case of Idiopathic Tetanus, Cured by Spirits of Turpentine

**Published:** 1842-12-03

**Authors:** Q. Gibbon

**Affiliations:** Salem, (N. J.)


### MEDICAL EXAMINER.

NEW SERIES.

No. 49.] PHILADELPHIA, DECEMBER 3, 1842. [Vol. I.

### *Case of Idiopathic Tetanus cured by Spirits of Turpentine.*

By Q. Gibbon, M. D.

To the Editors of the Medical Examiner.

Gentlemen,—I believe the subjoined case will go far to sustain the much reputed powers of turpentine in Idiopathic Tetanus.

Charles, son of James S., aged about four years, was seized with stiffness of the lower jaw, September 26th, 1842.

Sept. 27th. Mouth open to the extent of one-fourth of an inch ; jaws immoveably fixed in that position ; rigidity of the abdominal muscles and of the inferior extremities, accompanied by pain upon pressure or motion ; occasional spasms, during which the head was thrown back as in opisthotonos ; no fever; no cerebral derangement ; bowels obstinately constipated ; spine free from tenderness ; arms unaffected.

Under the impression that worms might have something to do with the above train of symptoms, a full dose of spigelia, followed by castor oil and spt. terebinth., was given.

28th. The purgative had operated briskly, but brought away no worms ; no amendment. Ordered calomel 2 grs., opium $\frac{1}{2}$ gr., every two hours, until sleep should be induced. Warm-bath for half an hour night and morning, and fomentations of hot turpentine to the spine.

29th. No sleep ; no amendment save a slight relaxation of the jaw. Continue as yesterday, and apply a blister to the spine.

30th. Still no sleep. Castor oil ℥j ; spts. terebinth. ʒii ; after the operation of which give laudanum twenty-five drops every three hours.

Oct. 1st. Purged freely. Laudanum has produced no narcosis whatever. Jaws somewhat relieved ; no other change. Laudanum by the teaspoonful every two hours until sleep should be induced ; length of the bath increased to one hour twice a day.

2d. Slight flush in the face ; occasional delirium ; opisthotonos to a considerable extent. Discontinue the laudanum, and substitute twenty-five drops spts. terebinth. with same quantity of tinct. assafœt. every four hours.

3d. Opisthotonos on the increase. Dr. Keasley saw the patient to-day, and recommended a teaspoonful of the turpentine every two hours.

4th. No change. Take a teaspoonful of the turpentine every hour.

6th. Copious discharges of black fœcal matter ; slight relaxation while in bath. Continue the turpentine as before.

7th. Black stools continue ; further relaxation while in the bath, much exhaustion from the latter. Continue the turpentine and use the bath but once a day.

8th. Evident amendment ; limbs more relieved ; sleep ; stools not so black ; nausea from the turpentine ; give one teaspoonful every two hours.

9th. Slept better ; rigidity less ; appetite for the first time ; stomach still rejects the turpentine. Discontinue it, and substitute small doses of laudanum.

11th. Rigidity much diminished; appetite improved; has slept soundly. From this period the boy slowly recovered under the use of small doses of laudanum and the daily use of the bath.

*Remarks.*—The above case presents some peculiarities worthy of note. The reader will be surprised at the large amount of calomel and opium administered, and their almost entire nullity of effect. No ptyalism or other effect from the calomel was perceived, and but a temporary flush and delirium from the opium and laudanum. I am induced to believe from inquiry of the mother, that the child took in the course of four days about forty grains of calomel fifteen grains of opium and fifteen teaspoonfuls of laudanum; and yet under these large doses the patient grew worse, the opisthotonos which was at first paroxysmal, becoming permanent. The appearance of the copious black fœtid discharges upon the fourth day from the first administration of the turpentine, seemed to be the signal for the abatement of the tetanic symptoms.

*Salem*, (N. J.) Nov. 24th, 1842.

